# Awareness and Use of Home-Based Respiratory Pathogen Testing Services in the Internet Era: Postpandemic Questionnaire Study

**DOI:** 10.2196/83767

**Published:** 2026-01-22

**Authors:** Chunshan Xu, Wenhao Cao, Cunbo Jia, Rongling Zhang, Ning Hu, Zhongguang Yu

**Affiliations:** 1 School of Management, Beijing University of Chinese Medicine, Beijing, China; 2 Department of Pulmonary and Critical Care Medicine, Renmin Hospital of Wuhan University, Wuhan, Hubei, China; 3 Respiratory Centre, China–Japan Friendship Hospital, No. 2, Yinghuayuan East Street, Chaoyang District, Beijing, 100029, China, 86 84206468; 4 China–Japan Friendship Hospital (Institute of Clinical Medical Sciences), Chinese Academy of Medical Sciences & Peking Union Medical College, Beijing, Beijing, China; 5 School of Population and Health, Renmin University of China, Beijing, China; 6 Economics and Management School, Wuhan University, Wuhan, China

**Keywords:** home-based testing, internet-based health care, respiratory pathogens, technology acceptance, testing

## Abstract

**Background:**

Home-based respiratory pathogen testing services (HRPTS), an emerging internet-based health care model, enable rapid pathogen identification within hours through digital platforms and eCommerce logistics. This decentralized approach overcomes conventional testing delays to accelerate diagnosis. However, public awareness, adoption, and influencing factors remain largely unknown.

**Objective:**

In this study, we aimed to investigate digitally connected metropolitan residents' awareness and intention to adopt HRPTS and analyze factors influencing adoption intention.

**Methods:**

This study used a structured questionnaire grounded in the technology acceptance model, which measured perceived usefulness, ease of use, risk, and behavioral intention. Questionnaire development involved focus group discussions to ensure content validity. Statistical analysis included descriptive statistics and multivariate linear regression, with scale reliability and validity confirmed by exploratory factor analysis. Using a convenience sampling strategy, 1850 volunteers completed questionnaires via Wenjuanxing. After data validation, 1756 surveys met the inclusion criteria (effective response rate: 94.92%) and were analyzed.

**Results:**

Among 1756 respondents, 54.7% (n=961) knew about HRPTS for respiratory diseases, and 15.3% (n=269) had previously used them. Perceived usefulness was high among respondents: fast pathogen identification (n=1092, 62.2%), early treatment (n=1136, 64.7%), time or cost savings (n=1119, 63.7%), and anxiety alleviation (n=1110, 63.2%). Regarding perceived ease of use, 55.9% (n=982) of the respondents cited robust logistics, 53.8% (n=945) cited online appointment convenience, and 54.2% (n=952) cited simple self-sampling. However, respondents expressed concerns regarding privacy (n=925, 52.7%), test accuracy questions (n=871, 49.6%), and insufficient regulations (n=948, 54.0%). Nevertheless, >70% of the respondents were willing to adopt HRPTS, if available. Multivariate regression showed that higher education (*β*=.598; *P*<.001), living with family (*β*=.271; *P*=.04), and absence of underlying chronic diseases (*β*=.321; *P*=.03) were significant predictors of adoption intention. Additionally, not having used HRPTS before (*β*=−1.203; *P*<.001) and less frequent health care–seeking behaviors were negatively associated with adoption intention.

**Conclusions:**

HRPTS as an internet-based health care service holds value for early diagnosis, treatment, and health care optimization in urban China. However, significant concerns regarding test accuracy, data privacy, and regulatory accountability within this evolving digital health sector should be addressed to strengthen respiratory disease prevention in the postpandemic era.

## Introduction

Respiratory infectious diseases are contagious and rapidly spreading illnesses caused by pathogens (eg, viruses and bacteria) transmitted through the respiratory tract. These conditions, characterized by high transmissibility and rapid spread, adversely impair global public health and provoke wider socioeconomic consequences [1-3]. For instance, seasonal influenza imposes substantial global burdens annually, causing 4‐23 million hospitalization cases of lower respiratory tract infections and 290,000‐650,000 seasonal influenza-associated respiratory deaths [4]. During the COVID-19 pandemic, 760 million cases were reported worldwide, with 6.81 million deaths [5]. Given the high pathogenicity and transmissibility of respiratory infectious diseases, accurate pathogen testing is essential to interrupt transmission chains and reduce the disease burden.

Advances in medical technology have facilitated the widespread adoption of respiratory pathogen testing, which enhances efficient identification for accurate early diagnosis and treatment [6] and optimizes clinical decision-making and therapy planning [7,8]. Current detection approaches for respiratory pathogens include at-home antigen detection and polymerase chain reaction (PCR) tests in hospital settings and clinical laboratories. Although antigen detection tests offer rapid results, they exhibit lower sensitivity [9,10] and carry a high risk of false-negative results, thereby necessitating frequent confirmatory testing [10,11]. The PCR test is a gold standard of respiratory pathogen testing owing to its high accuracy, broad detection spectrum, and standard operating procedures [12-15]. However, it depends heavily on clinical laboratories and requires patients to navigate a multistep and lengthy process from appointment scheduling and sampling to results waiting [16]. Moreover, during seasonal influenza or pandemic outbreaks, crowded hospitals face dual challenges: higher cross-infection risks in crowded waiting areas [17,18] and potential hospital congestion [16]. These critical limitations hinder rapid diagnosis and effective containment of respiratory diseases.

Therefore, in the postpandemic era, eCommerce platforms have pioneered the home-based respiratory pathogen testing service (HRPTS), leveraging “proactive screening” awareness developed by frequent COVID-19 nucleic acid tests and the extensive internet network and efficient logistics systems in China. The model operates via internet-based health care platforms with three core components: (1) an online booking system for users to select among 12 testing items (eg, influenza virus, SARS-CoV-2, *Mycoplasma pneumoniae*), schedule flexible home-collection hours, and follow standardized sampling instructions; (2) a digital logistics system, based on intelligent scheduling algorithms, integrated with logistics personnel or local couriers to deliver samples, thereby ensuring biosafety in transportation; and (3) a telereporting platform delivering reports to users within 3 hours of laboratories’ conducting nucleic acid extraction, amplification, and data analysis using PCR and other testing technologies. It is important to note that, as an emerging service, HRPTS availability is primarily concentrated in major metropolitan areas of China, leveraging existing dense logistics networks. Compared with conventional testing, HRPTS transcends barriers of time and space owing to its three advantages: (1) patients can finish self-sampling to reduce cross-infection risks, instead of registering at hospitals for sampling; (2) a quick response mechanism with a 3-hour turnaround time dramatically improves diagnostic efficiency, particularly for mass screening during influenza seasons or pandemic emergencies; and (3) HRPTS supports public health authorities in optimizing methods of prevention and control by offering pathogen prevalence analysis in regions.

Although the HRPTS demonstrates considerable potential for rapid respiratory disease diagnosis, it is available only in certain first-tier cities in China. Public awareness regarding the HRPTS consequently needs to be increased; however, its adoption intention may be hindered by factors such as accuracy concerns, privacy issues, and limited accessibility [19,20]. Therefore, we aimed to investigate HRPTS value, convenience, and ease of use from the consumers’ perspective in first-tier cities to determine their genuine adoption intentions and concerns. Additionally, we aimed to explore how individual characteristics (eg, age, education, and health literacy) influence its adoption. This study addresses research gaps in this specific urban context by improving the framework of public adoption of the HRPTS and provides empirical evidence for policymakers and service providers in metropolitan areas to optimize HRPTS implementation and regulation.

## Methods

### Questionnaire Design

The questionnaire items were drawn from validated scales used in prior studies based on the technology acceptance model (TAM) framework [21] and were adapted to fit the context of HRPTS adoption. The TAM framework focuses on perceived usefulness (PU) and perceived ease of use (PEOU) to explain and predict users’ acceptance of a new technology. To ensure the constructs were clearly defined and relevant to the HRPTS context, we operationalized them as follows: PU was measured through items capturing its clinical and practical benefits (eg, accelerating pathogen testing and enabling early intervention). PEOU assessed the accessibility and simplicity of the service process. Perceived risk (PR) was explicitly tied to salient concerns in home-based testing, including test accuracy, data privacy, and regulatory safeguards. We extended the TAM by incorporating variables, such as PR (eg, privacy concerns and test reliability), to comprehensively evaluate service acceptance. To ensure the content validity and contextual appropriateness of the questionnaire, we convened a panel of 12 authoritative experts—including public health researchers, clinicians, and patient advocates—and employed a focus group discussion to screen and refine the questionnaire items. After discussing service adoption scenarios, potential barriers, and privacy concerns, the final questionnaire comprised 31 items, assessing demographic information (13 items), and the 18 items of the TAM scale, which covered PU (6 items), PR (5 items), PEOU (4 items), and behavioral intention (BI; 3 items). While our measures are derived from the TAM framework, our analytical objective differs from traditional model testing. We do not examine the structural relationships among TAM constructs (eg, the effect of PU on BI). Instead, we treat PU, PEOU, PR, and BI as separate outcome variables in regression models to directly assess how demographic factors influence each dimension. This approach aligns with our goal of identifying population-specific determinants for targeted interventions.

### Questionnaire Validation

We collected 300 pretest responses to assess the reliability and construct validity. Exploratory factor analysis using principal component analysis with varimax rotation was performed. The Kaiser-Meyer-Olkin measure was 0.91, and the Bartlett test of sphericity was significant (*χ*²_153_=4389.2; *P*<.001), confirming that the data were suitable for factor analysis. Exploratory factor analysis extracted 4 factors with eigenvalues greater than 1, which together explained 73.69% of the total variance (see Table S1 in Multimedia Appendix 1 for detailed variance explained). All items exhibited factor loadings greater than 0.64 on their theoretically expected factors, with no substantial cross-loadings (>0.40) on other factors (see Table S2 in Multimedia Appendix 1 for the complete factor loading matrix). Reliability was assessed using Cronbach α for each construct: PU (*α*=0.915), PR (*α*=0.927), PEOU (*α*=0.884), and BI (*α*=0.888), indicating excellent internal consistency. The overall scale reliability was 0.850. These results support the strong psychometric properties of the adapted TAM scale in the HRPTS context.

### Questionnaire Survey and Implementation

This web-based survey assessed data collected using WenJuanxing, a mainstream online survey platform. The survey primarily targeted digitally connected residents of major Chinese metropolises (ie, Beijing, Shanghai, Guangzhou, and Shenzhen), supplemented by a smaller cohort from other provinces. This sampling strategy aimed to capture the perceptions of populations with access to and familiarity with internet-based services. Data collection occurred over a 12-day period (January 17‐28, 2024). This period was deliberately chosen to coincide with a peak influenza season, as HRPTS is most relevant and its perceived value is most salient when respiratory infection prevalence is high. While this may limit generalizability to nonseasonal periods, it allows us to capture adoption intentions under conditions of high practical relevance. After excluding invalid responses (ie, responses with completion times of less than 60 seconds, which were considered inattentive or insincere) and respondents <18 years old (n=94), we retained 1756 valid samples, with a valid response rate of 94.92%. This procedure yielded 1329 responses from first-tier cities and 427 from provincial areas.

### Ethical Considerations

This study strictly adhered to the World Medical Association Declaration of Helsinki and was approved by the Ethics Committee of China–Japan Friendship Hospital (approval no. 2024-KY-254). The research was conducted anonymously, and no personally identifiable information was collected. All respondents provided informed consent; for this online survey, consent was obtained electronically where participants indicated their agreement to participate after reading a detailed information page that outlined the study purpose, risks, benefits, and data confidentiality measures. Participants were informed that their data would be used solely for academic research purposes, and all questionnaire submissions were fully anonymized. As a token of appreciation, each participant received an electronic red packet reward of 2 RMB (US $0.3) upon completion of the survey.

### Statistical Analysis

Preliminary data management was conducted using Microsoft Excel 2021. Descriptive statistics were analyzed with SPSS Statistics 22 to summarize demographic characteristics and core TAM variables (PU, PR, PEOU, BI).

We used multivariate linear regression to examine factors influencing HRPTS adoption. The dependent variables were continuous composite scores for PU, PR, PEOU, and BI, calculated by summing responses to all items within each multi-item construct. Summing Likert-scale items to create composite scores is a common practice that approximates continuous data suitable for linear regression. Categorical predictors were converted into dummy variables for analysis. The coding scheme and reference categories were as follows: Sex: 0=Male (reference), 1=Female; Age: 0=<60 years (reference), 1=≥60 years; Education: 0=Less than bachelor’s degree (reference), 1=Bachelor’s degree or higher; Living arrangement: 0=Lives alone (reference), 1=Cohabiting with others; Previous respiratory infection: 0=Yes (reference), 1=No; Previous HRPTS use: 0=Yes (reference), 1=No; Health care–seeking behavior: A 3-category variable entered as 2 dummy variables, with “Always seek care” as the reference; Underlying chronic diseases: 0=Yes (reference), 1=No. Due to the mandatory-response setting on the online survey platform (Wenjuanxing), there were no missing data for the key variables included in the regression analyses.

Prior to interpretation, key regression assumptions were validated. Visual inspection of histograms, Q-Q plots, and residual scatterplots indicated no severe violations of normality or homoscedasticity. All variance inflation factor values were below 1.5 for individual predictors, confirming the absence of substantial multicollinearity, and Durbin-Watson statistics (range: 1.88‐1.93) suggested independent residuals. Detailed model fit indices (*R*², adjusted *R*², F-statistic, Durbin-Watson, variance inflation factor) are reported in Table S3 in Multimedia Appendix 1. The results are presented comprehensively in the supplementary materials: Table S2 in Multimedia Appendix 1 reports the unstandardized coefficients (β) with 95% CIs and *P* values, while Table S3 in Multimedia Appendix 1 additionally provides the standardized coefficients (β) to facilitate the comparison of the relative strength of predictors within and across models.

## Results

### Respondent Information

As shown in Table 1, among 1756 individuals, most (n=1064, 60.6%) respondents were female, and 31.1% (n=546) were aged 30‐39 years. Bachelor’s degree holders formed the largest group at 40.3% (n=708), followed by individuals with a high school education or less at 26.8% (n=470), and individuals with an associate degree at 21.8% (n=383). Additionally, 70.0% (n=1229) of the respondents cohabited with family members, of whom 56.8% (n=698) lived with older family members and 62.3% (n=766) lived with their children. In addition, 173 individuals, representing 14.1% of the cohabiting respondents, resided with family members with underlying diseases. Moreover, 448 (25.5%) respondents reported hypertension, diabetes, or other underlying diseases. When experiencing cold or influenza-like symptoms (eg, fever, cough, sore throat, and nasal congestion), 448 (25.5%) respondents consistently sought medical care, whereas 326 (18.6%) opted not to visit health care facilities. The remaining 982 (55.9%) indicated that their decisions depended on their condition.

**Table 1.** Respondent information.

Variable	Response, n (%)
Sex	
Male	692 (39.4)
Female	1064 (60.6)
Age (y)	
18‐29	345 (19.7)
30‐39	546 (31.1)
40‐49	332 (19.0)
50‐59	142 (8.1)
60‐69	393 (22.4)
70‐79	108 (6.2)
80‐90	10 (0.6)
Education	
High school or less	470 (26.8)
Associate degree	383 (21.8)
Bachelor’s degree	708 (40.3)
Master’s degree	156 (8.9)
Doctoral degree	39 (2.2)
Habitation pattern	
Lives alone	408 (23.2)
Cohabiting with family	1229 (70.0)
Cohabiting with roommates	91 (5.2)
Cohabiting with strangers	28 (1.6)
Cohabitant type (multiple responses)	
Elderly	698 (56.8)
Children	766 (62.3)
Pregnant women	36 (2.9)
Family members with underlying chronic medical conditions	173 (14.1)
Other	143 (11.6)
Have underlying chronic diseases	
Yes	448 (25.5)
No	1308 (74.5)
Type of underlying chronic diseases (multiple responses)	
Hypertension	238 (53.1)
Diabetes	185 (41.3)
COPD	84 (18.8)
Coronary heart disease	112 (25.0)
Cancer	71 (15.9)
Other	37 (8.3)
Respiratory pathogen infection (eg, influenza, SARS-CoV-2) in the previous year	
Yes	816 (46.5)
No	940 (53.5)
Health care–seeking frequency for cold or influenza-like symptoms	
Always	448 (25.5)
Depending on the condition	982 (55.9)
Never	326 (18.6)
Familiarity with HRPTS^a^	
Very familiar (detailed knowledge)	259 (14.8)
Familiar (core tests/process)	285 (16.2)
Moderately familiar (service awareness)	417 (23.8)
Slightly aware (vague recognition)	307 (17.5)
Unaware (no exposure)	488 (27.8)
Previous use of HRPTS	
Yes	269 (15.3)
No	1487 (84.7)
HRPTS recipient (multiple responses)	
Self	170 (63.2)
Elderly family member	115 (42.8)
Children	119 (44.2)
Other family member	23 (8.6)
Symptoms triggering HRPTS consideration (multiple responses)	
Dyspnea/chest tightness	956 (54.4)
Fever	530 (30.2)
Cough	1145 (65.2)
Myalgia	351 (20.0)
Headache	250 (14.2)
Sore throat	364 (20.7)
Nasal congestion	430 (24.5)

a HRPTS: home-based respiratory pathogen testing service.

A total of 961 (54.7%) respondents were aware of HRPTS. However, among all respondents, only 259 (14.8%) were very familiar with the detailed information and procedures, whereas 488 (27.8%) had never heard of HRPTS. Among 269 (15.3%) previous users, 170 (63.2%) purchased the service for personal use, while 115 (42.8%) and 119 (44.2%) used the service for their elderly family members and children, respectively. Additionally, more than half of the respondents considered using the HRPTS when experiencing chest tightness or dyspnea (n=1145, 65.2%) or fever symptoms (n=956, 54.4%), followed by cough (n=530, 30.2%) and myalgia (n=430, 24.5%).

### HRPTS Public Adoption Intention

**Figure 1.**
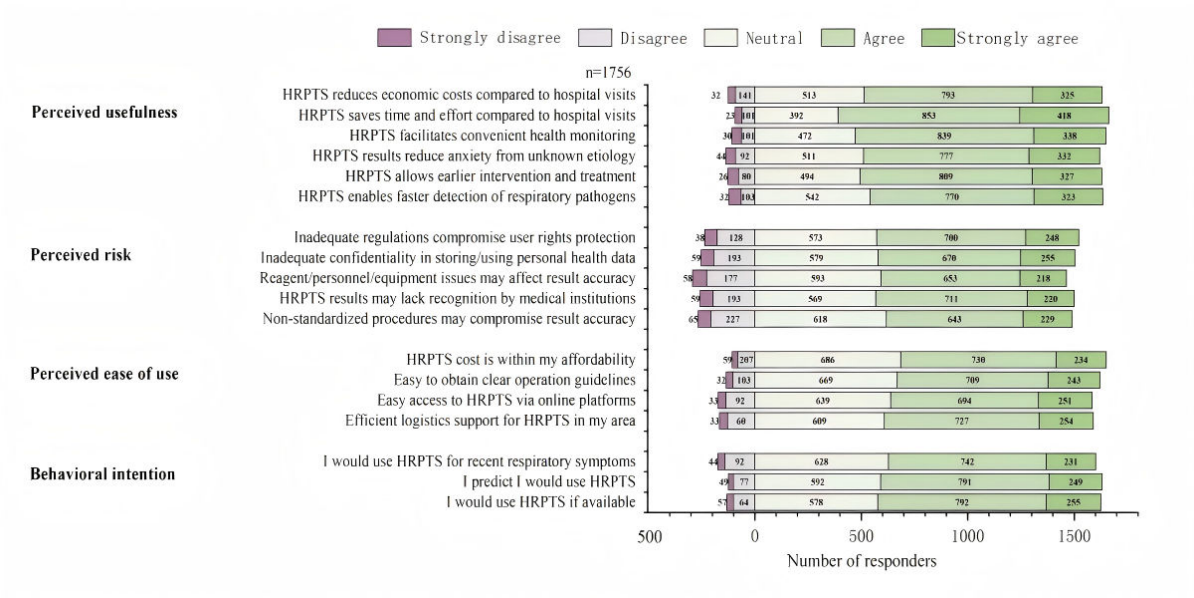
Home-based respiratory pathogen testing services (HRPTS) adoption intention survey.

We assessed HRPTS perceptions across 4 dimensions using a structured questionnaire: PU, PR, PEOU, and BI. The detailed results are presented in Figure 1.

Most participants agreed that the HRPTS promoted rapid pathogen identification (770 [43.8%] agreed; 323 [18.4%] strongly agreed) and early intervention (809 [46.1%] agreed; 327 [18.6%] strongly agreed). The HRPTS also reduced anxiety caused by undiagnosed symptoms (777 [44.2%] agreed; 332 [18.9%] strongly agreed) and facilitated family health monitoring (839 [47.8%] agreed; 338 [19.2%] strongly agreed). However, 93 (5.3%) individuals disagreed that the HRPTS saved time and energy, and 1118 (63.7%) individuals agreed or strongly agreed that it reduced costs.

Additionally, more than half of the respondents thought that robust local logistics could support the HRPTS (727 [41.4%] agreed; 254 [14.5%] strongly agreed), and accessing the HRPTS on online platforms was easy (694 [39.5%] agreed; 251 [14.3%] strongly agreed). Moreover, most participants suggested that they could effortlessly obtain clear and easy-to-follow instructions (709 [40.4%] agreed; 243 [13.8%] strongly agreed). Among the participants, 964 (54.9%) considered the HRPTS affordable, 686 (39.1%) remained neutral, and 106 (6.0%) found it expensive.

Substantial concerns have emerged about the HRPTS’s potential risks, despite its high ratings for PU and PEOU. When evaluating the challenges associated with HRPTS adoption, respondents expressed strong agreement on 2 primary issues: 40.5% (n=711) agreed and 12.5% (n=220) strongly agreed that test results may not be recognized by medical institutions, whereas 39.9% (n=700) agreed and 14.1% (n=248) strongly agreed that existing inadequate regulations on HRPTS could not guarantee their rights. Moreover, 37.2% (n=653) agreed and 12.4% (n=218) strongly agreed on reliability concerns regarding detection reagents, personnel, and equipment; 36.6% (n=643) agreed and 13.0% (n=229) strongly agreed that improper operations could compromise test accuracy; and 38.2% (n=670) agreed and 14.5% (n=255) strongly agreed on safeguards for data privacy.

Therefore, we further assessed BI to use the HRPTS after understanding the respondents’ awareness. Most expressed a willingness to adopt an HRPTS if it were available in the market (792 [45.1%] agreed; 255 [14.5%] strongly agreed), a few refused to adopt it (23 [1.3%] strongly disagreed; 101 [5.8%] disagreed), and 585 (33.3%) remained neutral. Additionally, 742 (42.3%) individuals agreed and 231 (13.2%) strongly agreed that they would use the HRPTS for pathogen testing if experiencing recent respiratory infection symptoms.

### Analysis of Individual Characteristics Influencing HRPTS Adoption

Multivariate linear regression analysis was used to examine the associations between 8 demographic characteristics (eg, sex, age, and education) and health behaviors and the HRPTS adoption intention (Table 2). The results showed the differential effects of these factors on PU, PR, PEOU, and BI. The respondents were subsequently grouped, based on age, using a threshold of 60 years and education (ie, nontertiary education [associate degree, high school or less] vs tertiary education [bachelor’s, master’s, or doctoral degree]).

**Table 2.** Linear regression analysis on multiple factors influencing home-based respiratory pathogen testing services (HRPTS) adoption intention.

Variable	PU^a^			PR^b^			PEOU^c^			BI^d^		
	*β*	95% CI	*P* value	*β*	95% CI	*P* value	*β*	95% CI	*P* value	*β*	95% CI	*P* value
Sex												
Male	Reference			Reference			Reference			Reference		
Female	.755	0.361 to 1.194	.001	−.058	−0.472 to 0.355	.78	.142	−0.139 to 0.423	.32	.066	−0.157 to 0.289	.56
Age (y)												
<60	Reference			Reference			Reference			Reference		
≥60	−.594	−1.204 to −0.016	.06	−.59	−1.164 to −0.016	.04	−.412	−0.802 to −0.023	.04	−.242	−0.552 to 0.068	.13
Education												
Less than a bachelor’s degree	Reference			Reference			Reference			Reference		
Bachelor’s degree or higher	.887	0.404 to 1.370	<.001	.774	0.320 to 1.229	.001	.519	0.210 to 0.827	.001	.598	0.353 to 0.844	<.001
Living arrangement												
Lives alone	Reference			Reference			Reference			Reference		
Cohabiting with others	.611	0.107 to 1.116	.02	−.089	−0.565 to 0.386	.71	.371	0.049 to 0.694	.02	.271	0.014 to 0.527	.04
Previous respiratory pathogen infection (within 1 y)												
Yes	Reference			Reference			Reference			Reference		
No	−.211	−0.659 to 0.237	.36	−.233	−0.655 to 0.189	.28	.038	−0.249 to 0.325	.80	.052	−0.176 to 0.280	.65
HRPTS utilization												
Yes	Reference			Reference			Reference			Reference		
No	−.831	−1.482 to −0.180	.01	−1.266	−1.879 to −0.653	＜.001	−1.596	−2.012 to −1.179	＜.001	−1.203	−1.534 to −0.872	<.001
Health care–seeking for Influenza-like symptoms												
Always seek care	Reference			Reference			Reference			Reference		
Seek care depending on context	−1.002	−1.547 to −0.457	<.001	.102	−0.411 to 0.615	.70	−.384	−0.732 to −0.036	.03	−.411	−0.688 to −0.134	.004
Never seek care	−1.221	−1.919 to −0.522	.001	.072	−0.586 to 0.730	.83	−.553	−1.000 to −0.107	.02	−.609	−0.965 to −0.254	.001
Have underlying chronic diseases												
Yes	Reference			Reference			Reference			Reference		
No	.506	−0.64 to 1.076	.08	−.09	−0.627 to 0.446	0.74	.376	0.011 to 0.740	0.04	.321	0.031 to 0.611	.03

a PU: perceived usefulness.

b PR: perceived risk.

c PEOU: perceived ease of use.

d BI: behavioral intention.

Multivariate regression analysis revealed significant associations between sex, education, habitation patterns, previous usage, and health care–seeking behaviors with HRPTS-PU. Compared with men, women had higher ratings for PU (*β*=.755, *P*=.001). Respondents with a bachelor’s or higher degree perceived greater usefulness than less educated respondents (*β*=.887, *P*<.001). Cohabiting individuals perceived higher usefulness than individuals living alone (*β*=.611, *P*=.09). Nonusers reported lower PU than previous users (*β*=–.831, *P*=.01). Additionally, stratified statistics on health care–seeking behavior revealed a stepwise decline in PU: relative to the “always seeking care” subgroup, the “occasionally seeking care” (*β*=–1.002, *P<*.001) and “never seeking care” (*β*=–1.221, *P*=.001) subgroups showed progressively lower PU.

The main factors associated with PR were age, education, and previous usage. Age had a negative association with respondents aged ≥60 years showing lower PR (*β*=–.59, *P*=.04). By contrast, individuals with tertiary education were correlated with a higher PR against individuals with lower education (*β*=.774, *P*=.001). HRPTS previous users reported lower PR than nonusers (*β*=–1.266, *P<*.001).

PEOU was associated with age, education level, and underlying disease status, among other factors. Older adults (aged ≥60 y) had a negative association (*β*=–.412, *P*=.04); cohabiting individuals perceived a greater PEOU than individuals living alone (*β*=.371, *P*=.024). Individuals with tertiary education were associated with better ease-of-use evaluations than less-educated individuals (*β*=.519, *P*=.001). Service experiences showed significant effects, with nonusers reporting a precipitous decline in PEOU (*β*=–1.596, *P<*.001). Individuals without underlying diseases reported more positive ease-of-use perceptions than individuals with diseases (*β*=.376, *P*=.043).

Thus, education level, previous usage, and health care–seeking behaviors were markedly associated with BI to use the HRPTS. Individuals with tertiary education predicted stronger adoption intention (*β*=.598, *P<*.001). Stratification of health care–seeking behaviors revealed that, compared with the “always seeking care” subgroup, the “occasionally seeking care” (*β*=–.411, *P*=.004) and “never seeking care” (*β*=–.609, *P*=.001) subgroups had progressively lower BI. Additionally, nonusers exhibited lower BI (*β*=–1.203, *P<*.001). Cohabitation (*β*=.271, *P*=.039) and absence of underlying diseases (*β*=.321, *P*=.03) also positively influenced adoption intention.

## Discussion

This study uses the constructs of the TAM as a conceptual foundation but adopts a distinct analytical focus. Rather than testing the structural pathways within TAM (eg, the effects of PU and PEOU on BI) in the current analysis, we treat its core dimensions—PU, PEOU, PR, and BI—as separate outcomes. Future research will explicitly examine these interconstruct relationships to elucidate the pathways to adoption. This present approach allows us to directly examine how demographic and health-behavioral factors influence each dimension, facilitating the identification of key subgroups for tailored strategies.

The aim of this study was to investigate the awareness and intention to adopt HRPTS among residents in major Chinese metropolitan areas and analyze factors influencing adoption intention. Our findings, reflecting the attitudes of a predominantly young, educated, and digitally literate urban sample, indicate that more than half of the respondents were aware of the HRPTS for respiratory diseases and 15.3% (n=269) had previously used it; factors significantly associated with a stronger adoption intention included higher education level, living with family, and absence of underlying chronic diseases. Previous non-use of HRPTS and less frequent health care–seeking behaviors were associated with lower adoption intention. The HRPTS provides multiple benefits for disease prevention, treatment, and public health. First, the HRPTS accelerated pathogen identification, as acknowledged by 62.2% (n=1092) of the respondents. Additionally, when users manifest respiratory symptoms, such as fever or cough, they can bypass hospital visits by scheduling an HRPTS through a mobile app. Test reports covering 12 common pathogens (eg, influenza virus, SARS-CoV-2, and *M pneumoniae*) are therefore produced within 3 hour, allowing prompt identification. This process effectively prevents a condition from worsening due to delaying treatment and facilitates early identification while supporting public health interventions. Moreover, 64.7% (n=1136) of the participants recognized its role in early interventions because targeted antiviral drugs (eg, optimal oseltamivir administration within 48 hours against influenza A [17,22,23]) are administered after pathogen identification, thereby reducing antibiotic misuse and improving efficacy. Furthermore, this approach provides particular clinical value for high-risk groups, such as children and older adults, thereby preventing progression to severe complications (eg, pneumonia and respiratory failure).

Second, the HRPTS provides significant economic benefits. In conventional health care, patients incur direct costs (eg, transportation and registration fees), in addition to the time and effort required for queuing. Based on our findings, 63.67% (n=1118) of the respondents recognized the cost reduction effects of the HRPTS and 72.4%（n=1271） acknowledged its time-saving advantages. Additionally, during respiratory disease outbreaks (eg, influenza seasons and pandemics) [24,25], tertiary hospitals frequently experience limited medical resources, leading to lower care efficiency and higher cross-infection risks. The HRPTS was designed to leverage internet-based technologies to address this crisis through decentralized community- or home-based testing. By enabling remote sample collection coordination and digital result delivery, it efficiently redistributes patient flows, alleviates institutional strain, and optimizes medical resource allocation.

Third, the HRPTS helps alleviate anxiety among patients and their families. When susceptible populations (eg, children, the elderly) develop respiratory symptoms, their family members often experience anxiety due to the difficulty of assessing illness severity [26,27]. For instance, 1 study [28] on respiratory tract infection in children reported a 39% reduction in the level of anxiety in their parents after a definitive diagnosis. Therefore, our results showed that 63.2% (n=170) of the respondents recognized the anxiety-relieving effects of the HRPTS through rapid pathogen identification, thereby reducing diagnostic uncertainty and providing a safer health care alternative that avoids hospital-acquired infection risks.

Fourth, while the convenience and ease of use of the HRPTS are critical determinants of public adoption, efficient logistics underpin the HRPTS end-to-end service chain (ie, collection–transportation–testing), ensuring timely sample delivery and reliable testing. Statistical analysis revealed that 55.9% (n=982) of the respondents reported accessible and efficient logistics support via mature networks (JD Logistics; Meituan Delivery), which enable a 3-hour average turnaround time in urban cores. Smart algorithms can further optimize routing (eg, avoiding midday congestion) [29,30] and “emergency supply chain for testing,” which is added during influenza seasons, ensures timely specimen delivery [31].

Fifth, handy digital platforms proved equally essential, with 53.8% (n=945) of the respondents confirming easy access to the HRPTS via these platforms. These platforms, serving as the primary interface for the HRPTS, automatically recommend appropriate testing panels to users and functions, such as appointment scheduling, logistics tracking, and results checking to enhance service accessibility and transparency. Evidence indicates that electronic appointment platforms substantially reduce efforts into offline coordination [32]. Furthermore, the HRPTS utilizes geolocation to identify the nearest testing facilities, allowing users to monitor real-time logistics and laboratory progress via mobile apps or online mini programs. These digital platforms consequently streamline test scheduling and result queries for patients, improve service accessibility and transparency, and deliver timely and efficient health care services to community members.

Finally, standardized sampling for the HRPTS reduces the processing time. Another previous study [33] reported that user error rates are positively correlated with procedural complexity. In this study, 54.2% (n=952) of the respondents reported receiving clear, easy-to-follow instructions, which enabled successful self-sampling with nasopharyngeal or oropharyngeal swabs and subsequent specimen transportation. Thus, well-designed procedures are pivotal for HRPTS adoption, featuring simple and easy-to-follow instructions that enable nonprofessionals to perform accurate self-sampling. Furthermore, the process incorporates biosafety reminders and standardized biosafety-compliant packaging.

Despite its convenience and benefits, the HRPTS has multiple risks. A primary concern regarding the HRPTS is data privacy breaches in its adoption, with 52.7% (n=925) of the respondents expressing concerns about the confidentiality of their personal information. These concerns stem from multistage data exposure risks from user registration (eg, names, addresses, and symptom complaints) to test results transmission (eg, pathogen types and linkages with electronic health records) because each stage involves the collection and sharing of sensitive health data. Data from a previous study [34] indicated that 32.5% of the health care data breaches between 2010 and 2020 resulted from cyberattacks or information technology incidents, primarily targeting decentralized services, such as home health monitoring platforms and telehealth diagnosis systems. Additionally, an empirical study by Smith [35] revealed that 19% of the medical data breaches originated from supply chain loopholes (eg, unencrypted logistics interfaces, contractor privilege misuse), with each incident affecting an average of 87,000 individuals. These findings underscore security issues in cross-institutional health data exchanges. Therefore, delivery terminals should not display users’ detailed addresses, and the security of the recorded video storage must be strictly controlled during front-end data collection. Moreover, all data transmissions should utilize encrypted channels, and expired data should be purged using established protocols. Clear boundaries should also be established for third-party data sharing to ensure complete anonymization of all shared data.

Testing accuracy is another major concern in HRPTS implementation, with nearly half of the respondents expressing apprehension about potential inaccuracies owing to user errors or technical limitations. This aligns with our operationalization of PR to explicitly include accuracy concerns. These concerns are primarily derived from potential issues in 2 key links: improper self-sampling practices and technical limitations of detection systems. For instance, common problems associated with self-swabbing from the nasopharynx include insufficient sampling depth and inadequate swabbing duration. Additionally, temperature fluctuations during specimen transportation may reduce viral viability to >50% of that observed under stable low-temperature conditions, whereas nucleic acid integrity can decrease by up to 40% [36]. Although these changes directly affect a test’s sensitivity, these concerns may be addressed because evidence indicates that self-collected throat and nasal specimens can achieve pathogen (or other analyte) detection accuracy comparable to that of samples obtained by health care workers [37]. In this regard, regulatory approval of the HRPTS indicates that its detection system has been certified by relevant authorities and complies with established standards. Nevertheless, government agencies should rigorously monitor laboratory certifications, ensuring that qualification reviews, operational procedures, and data management comply with regulations to further alleviate user concerns. This oversight is crucial to prevent enterprises from prioritizing efficiency over quality.

Finally, the lack of supportive policy frameworks is another major public concern, with over half of the respondents expressing apprehension regarding the insufficient HRPTS regulations and insufficient protection of users’ rights. Additionally, inconsistent recognition of third-party rapid testing results by medical institutions may lead to discrepancies in clinical decision-making [38]. For instance, patients may wonder whether inconsistent recognition of test results will trigger redundant tests, delays in treatment, and worsening resource depletion if hospitals dismiss test reports from other testing facilities. Furthermore, when issues such as sample contamination, misdiagnosis, or privacy breaches occur, identifying the liable party is difficult for any of the detection agencies, service personnel, or technology platform providers that could become the subject of disputes. Ambiguity also arises from the lack of detailed provisions for these emerging services, leaving consumers without a clear recourse. Therefore, defining the scope of the HRPTS validity, entry criteria for institutions, and dispute resolution mechanisms through administrative action is crucial.

A notable finding is the apparent gap between positive adoption intention (>70% willingness) and low actual usage (15.3%), coupled with a substantial proportion of neutral attitudes. This pattern suggests a “BI-action gap” commonly observed in the adoption of novel health technologies. Several contextual factors may explain this discrepancy. First, as an emerging service, HRPTS naturally faces initial public caution; awareness does not immediately translate into use. Second, the service’s limited geographic coverage (primarily first-tier cities) physically restricts access for many. Third, when experiencing actual symptoms, individuals may revert to habitual health care–seeking pathways (eg, hospitals) despite prior expressed interest in alternatives. Finally, the significant concerns regarding accuracy, privacy, and regulation, as identified in our study, likely act as critical psychological barriers that inhibit the conversion of positive intention into actual trial. Future longitudinal research is needed to track how adoption evolves with increased service maturity, publicity, and policy support.

This study revealed group differences in HRPTS adoption intention linked to interactions between individual characteristics and health behaviors. In particular, older participants aged ≥60 years demonstrated negative associations between PR and ease of use, which may reflect higher risk perception and lower adoption intention potentially related to limited digital literacy and health knowledge [39,40]. Community-based interventions and assistance from family doctors are consequently required to improve service accessibility for this population. Additionally, individuals living alone exhibited negative correlations between PU and BI, associated with insufficient social support [41]. Therefore, establishing robust social support networks may be important for encouraging proactive health management is imperative. Moreover, the “occasionally seeking care” and “never seeking care” subgroups exhibited negative attitudes toward service usefulness and BI, indicating lower acceptance rates. This finding suggests that low health motivation can dampen new service adoption, which is consistent with the health motivation component of the health belief model [6,42], and that primary care screening and health education interventions should be implemented to enhance engagement. Furthermore, individuals without underlying diseases showed notably stronger positive correlations between PU and BI, indicating that healthier individuals prefer convenient and efficient services and demonstrated a higher acceptance of new health care services. Thus, we propose a tiered implementation strategy comprising the following components: publicizing the service among populations with different education levels, optimizing procedures with a focus on user experience, offering directed support for susceptible groups such as elderly individuals and individuals living alone, and reinforcing public motivation by monitoring their health behaviors. This strategy aims to improve the social acceptance and efficacy of the HRPTS as a health service.

This study has several limitations. First, the generalizability of the findings is constrained by the sampling method and scope. Our online convenience sample predominantly represents younger, more highly educated, and digitally literate residents of major Chinese cities and may not reflect the attitudes of the general population, including older adults or those in rural areas. Second, the cross-sectional design captures perceptions at a single point in time and cannot establish causality or track evolution. Third, the context of data collection—a 12-day period during peak influenza season—while relevant for capturing high-demand scenarios, may limit extrapolation to other times of the year. Fourth, as an emerging service, HRPTS is currently self-paid and geographically limited, and its technical and policy frameworks are still evolving; thus, acceptance may shift with future developments. These factors should be considered when interpreting the results.

In conclusion, this study systematically elucidated the value, adoption determinants, and risks of the HRPTS among metropolitan residents in China. More than 60%（n=1054） of the respondents acknowledged its core value in accelerating pathogen identification, initiating early treatment, reducing economic burden and health care delays, and alleviating distress. Efficiency emerged as a critical adoption driver, with 55.9% (n=982) appreciating internet-facilitated logistics (eg, a 3-h turnaround time in urban cores), 53.8% (n=945) endorsing digital platform accessibility, and 54.2% (n=952) completing self-sampling using clear protocols. However, 3 key barriers were identified: 52.7% (n=925) of the respondents were concerned about privacy breaches, which require data encryption and supply chain security; approximately half of respondents questioned the accuracy of the results, highlighting the need to strengthen laboratory qualifications and sampling training; and half of respondents were concerned about the lack of policies, indicating the urgent need to clarify the service validity, access criteria, and dispute resolution mechanisms for the HRPTS. We predominantly evaluated the use of the HRPTS by residents in first-tier cities, highlighting its contributions to ensuring early diagnosis and treatment, improving clinical workflows, and redistributing strained medical resources through internet-mediated care pathways. However, our results also indicated that efforts must address challenges related to privacy concerns, testing accuracy, and regulation through technological optimization, policy improvement, and public education to help build a more resilient prevention and control system for respiratory diseases. Nevertheless, the HRPTS has broad prospects for application and promotion in first-tier cities and urban clusters across the world that are equipped with internet and logistics infrastructure and face limited medical resources or higher demand for convenient health care (eg, high-density Asian cities, Western metropolitan areas, and health care hubs across Africa and Latin America).

## Supplementary material

10.2196/83767 Multimedia Appendix 1 Supplementary material Tables S1-S3.

## Abbreviations

BI: behavioral intention
HRPTS: home-based respiratory pathogen testing service
PCR: polymerase chain reaction
PEOU: perceived ease of use
PR: perceived risk
PU: perceived usefulness
TAM: technology acceptance model
