# Supplementary material for: Awareness and Use of Home-Based Respiratory Pathogen Testing Services in the Internet Era: Postpandemic Questionnaire Study
Source: JMIR Form Res. 2026 Jan 22;10:e83767. doi: 10.2196/83767 (PMC12826645; doi:10.2196/83767)
Supplement: Multimedia Appendix 1 [file formative-v10-e83767-s001.docx]

****Supplementary Table S1. Reliability Coefficients and Total Variance Explained from EFA****

| ****A. Reliability Analysis (Cronbach‘s Alpha)**** |  |  |
| --- | --- | --- |
| ****Construct**** | ****No. of Items**** | ****Cronbach‘s α**** |
| Perceived Usefulness (PU) | 6 | 0.915 |
| Perceived Risk (PR) | 5 | 0.927 |
| Perceived Ease of Use (PEOU) | 4 | 0.884 |
| Behavioral Intention (BI) | 3 | 0.888 |

| **B. Total Variance Explained** | | | | | | | |
| --- | --- | --- | --- | --- | --- | --- | --- |
| **Component** | **Initial Eigenvalues** | | | | **Rotation Sums of Squared Loadings** | | |
|  | **Total** | **% of Variance** | **Cumulative %** | | **Total** | **% of Variance** | **Cumulative %** |
| 1 | 12.358 | 47.531 | 47.531 | | 5.65 | 21.73 | 21.73 |
| 2 | 3.582 | 13.778 | 61.309 | | 5.055 | 19.444 | 41.174 |
| 3 | 1.845 | 7.097 | 68.406 | | 4.521 | 17.39 | 58.564 |
| 4 | 1.373 | 5.28 | 73.686 | | 3.931 | 15.121 | 73.685 |
| *Extraction Method: Principal Component Analysis. | | | |  |  |  |  |

****Supplementary Table S2. Factor Loadings Matrix from Exploratory Factor Analysis (Varimax Rotation)****

| **Item** | **Description** | **PU** | **PR** | **PEOU** | **BI** |
| --- | --- | --- | --- | --- | --- |
| ****PU1**** | Reduces economic costs | ****0.809**** |  |  |  |
| ****PU2**** | Saves time and effort | ****0.846**** |  |  |  |
| ****PU3**** | Facilitates convenient health monitoring | ****0.740**** |  |  |  |
| ****PU4**** | Results reduce anxiety | ****0.799**** |  |  |  |
| ****PU5**** | Allows earlier intervention | ****0.681**** |  |  |  |
| ****PU6**** | Enables faster pathogen detection | ****0.756**** |  |  |  |
| ****PR1**** | Inadequate regulations compromise rights |  | ****0.772**** |  |  |
| ****PR2**** | Inadequate confidentiality of health data |  | ****0.877**** |  |  |
| ****PR3**** | Reagent/personnel issues affect accuracy |  | ****0.914**** |  |  |
| ****PR4**** | Results may lack medical recognition |  | ****0.873**** |  |  |
| ****PR5**** | Non-standardized procedures affect accuracy |  | ****0.845**** |  |  |
| ****PEOU1**** | Cost is affordable |  |  | ****0.807**** |  |
| ****PEOU2**** | Easy to obtain operation guidelines |  |  | ****0.831**** |  |
| ****PEOU3**** | Easy access via online platforms |  |  | ****0.739**** |  |
| ****PEOU4**** | Efficient logistics support |  |  | ****0.645**** |  |
| ****BI1**** | Would use for recent symptoms |  |  |  | ****0.784**** |
| ****BI2**** | Predict would use |  |  |  | ****0.761**** |
| ****BI3**** | Would use if available |  |  |  | ****0.733**** |

*Extraction Method: Principal Component Analysis. Rotation Method: Varimax with Kaiser Normalization. All cross-loadings were below 0.40. Rotation converged in 5 iterations.*

****Supplementary Table S3. Diagnostics and Standardized Coefficients for Multiple Linear Regression Models****

| **Model (Dependent Var.)** | **R²** | **Adj. R²** | **F (p-value)** | **Durbin-Watson** | **Max VIF** |
| --- | --- | --- | --- | --- | --- |
| Perceived Usefulness (PU) | 0.045 | 0.040 | 10.215 (p<0.001) | 1.891 | 1.445 |
| Perceived Risk (PR) | 0.030 | 0.025 | 6.711 (p<0.001) | 1.928 | 1.445 |
| Perceived Ease of Use (PEOU) | 0.063 | 0.059 | 14.801 (p<0.001) | 1.923 | 1.445 |
| Behavioral Intention (BI) | 0.070 | 0.066 | 16.508 (p<0.001) | 1.883 | 1.445 |

| ****Standardized Coefficients (Beta) by Predictor**** | ****PU**** | ****PR**** | ****PEOU**** | ****BI**** |
| --- | --- | --- | --- | --- |
| Sex (Female vs. Male) | ****0.080****** | -0.007 | 0.023 | 0.014 |
| Age (≥60 vs. <60) | -0.054 | ****-0.057***** | ****-0.058***** | -0.043 |
| Education (Bachelor+ vs. Lower) | ****0.096****** | ****0.090****** | ****0.087****** | ****0.126****** |
| Living arrangement (Cohabiting vs. Alone) | ****0.056***** | -0.009 | ****0.053***** | ****0.048***** |
| Previous Infection (No vs. Yes) | -0.023 | -0.027 | 0.006 | 0.011 |
| HRPTS Utilization (No vs. Yes) | ****-0.065***** | ****-0.106****** | ****-0.193****** | ****-0.183****** |
| Healthcare-seeking (Depending vs. Always) | ****-0.108****** | 0.012 | ****-0.064***** | ****-0.086****** |
| Healthcare-seeking (Never vs. Always) | ****-0.103****** | 0.007 | ****-0.072***** | ****-0.100****** |
| Underlying Diseases (No vs. Yes) | 0.048 | -0.009 | ****0.055***** | ****0.059***** |

*1.Coding Reference: See main text Statistical Analysis section for dummy variable coding details.

2.Diagnostics: VIF = Variance Inflation Factor. All individual predictor VIFs were below 1.5, confirming no multicollinearity. Durbin-Watson statistics close to 2 suggest independence of residuals.

3.Significance: *p<0.05, **p<0.01. Significance levels are based on the p-values of the unstandardized coefficients (see Supplementary Table S4 for details).
